# Characterization of Guinea Pig Antibody Responses to Salivary Proteins of *Triatoma infestans* for the Development of a Triatomine Exposure Marker

**DOI:** 10.1371/journal.pntd.0002783

**Published:** 2014-04-03

**Authors:** Veronika Dorňáková, Renzo Salazar-Sanchez, Katty Borrini-Mayori, Oscar Carrion-Navarro, Michael Z. Levy, Günter A. Schaub, Alexandra Schwarz

**Affiliations:** 1 Institute of Parasitology, Biology Centre of the Academy of Sciences of Czech Republic, Ceske Budejovice, Czech Republic; 2 Faculty of Science, University of South Bohemia, Ceske Budejovice, Czech Republic; 3 Universidad Peruana Cayetano Heredia, Sede de Arequipa, Arequipa, Peru; 4 Department of Biostatistics and Epidemiology, Center for Clinical Epidemiology and Biostatistics, School of Medicine, University of Pennsylvania, Philadelphia, Pennsylvania, United States of America; 5 Zoology/Parasitology Group, Ruhr-University Bochum, Bochum, Germany; IRD/CIRDES, Burkina Faso

## Abstract

**Background:**

Salivary proteins of *Triatoma infestans* elicit humoral immune responses in their vertebrate hosts. These immune responses indicate exposure to triatomines and thus can be a useful epidemiological tool to estimate triatomine infestation. In the present study, we analyzed antibody responses of guinea pigs to salivary antigens of different developmental stages of four *T. infestans* strains originating from domestic and/or peridomestic habitats in Argentina, Bolivia, Chile and Peru. We aimed to identify developmental stage- and strain-specific salivary antigens as potential markers of *T. infestans* exposure.

**Methodology and Principal Findings:**

In SDS-PAGE analysis of salivary proteins of *T. infestans* the banding pattern differed between developmental stages and strains of triatomines. Phenograms constructed from the salivary profiles separated nymphal instars, especially the 5^th^ instar, from adults. To analyze the influence of stage- and strain-specific differences in *T. infestans* saliva on the antibody response of guinea pigs, twenty-one guinea pigs were exposed to 5^th^ instar nymphs and/or adults of different *T. infestans* strains. Western blot analyses using sera of exposed guinea pigs revealed stage- and strain-specific variations in the humoral response of animals. In total, 27 and 17 different salivary proteins reacted with guinea pig sera using IgG and IgM antibodies, respectively. Despite all variations of recognized salivary antigens, an antigen of 35 kDa reacted with sera of almost all challenged guinea pigs.

**Conclusion:**

Salivary antigens are increasingly considered as an epidemiological tool to measure exposure to hematophagous arthropods, but developmental stage- and strain-specific variations in the saliva composition and the respective differences of immunogenicity are often neglected. Thus, the development of a triatomine exposure marker for surveillance studies after triatomine control campaigns requires detailed investigations. Our study resulted in the identification of a potential antigen as useful marker of *T. infestans* exposure.

## Author Summary

Chagas disease is caused by the protozoan parasite *Trypanosoma cruzi*, and currently affects approximately 8 million people in Latin American countries. Although vector control campaigns against the most effective Chagas disease vector, *Triatoma infestans*, have been highly successful, *T. infestans* is re-establishing in once-endemic regions. To monitor re-establishing triatomines, new epidemiological tools are needed. Antibody responses of hosts to triatomine salivary proteins represent a promising tool to detect biting bugs, and highly immunogenic salivary antigens may be used as markers of triatomine exposure. Therefore, we analyzed the antibody response of guinea pigs, common peridomestic hosts of *T. infestans*, to salivary proteins of nymphs and adults of four different *T. infestans* strains from Argentina, Bolivia, Chile and Peru. Developmental stage- and strain-specific proteins in the saliva of *T. infestans* influenced the antibody response of guinea pigs, and different salivary antigens were recognized by guinea pig sera. Despite the variations of immunogenic salivary antigens, a 35 kDa antigen was recognized by almost all guinea pig sera and this antigen may be a useful marker of *T. infestans* exposure.

## Introduction

Arthropod-borne diseases, such as malaria, leishmaniasis, Lyme disease and Chagas disease, greatly impact human and animal health worldwide [1]–[4]. For the improvement of vector control measures, much effort is being devoted to develop novel, simple, rapid and sensitive tools to monitor populations of hematophagous arthropods [5]–[8]. These tools may identify human beings and animals at risk of exposure to vector bites and parasite infection. A promising, immunological approach is based on the immunogenicity of salivary proteins from hematophagous arthropods. Salivary proteins of these arthropods are injected into their hosts while blood-feeding to counteract the vertebrate's hemostasis, inflammation, and immunity [9]–[11]. In vertebrates salivary proteins induce a humoral immune response, amongst others, and these antibody responses have been used to identify highly immunogenic salivary proteins that can serve as an immunological tool such as markers of exposure to arthropod bites [12].

Schwartz et al. [13] studied, as one of the first researchers, the relationship between arthropod exposure and antibody level. They discovered that outdoor workers who had been exposed to tick bites of *Ixodes dammini* had higher anti-saliva IgG antibody levels compared to workers that had not been exposed to ticks. Following these findings, several other studies characterized antibody responses of different animals to the saliva of hematophagous arthropods such as sand flies [e.g. 14–16], mosquitoes [e.g. 17,18], ticks [e.g. 19–21] and black flies [22], [23]. Furthermore, antibody responses of humans and/or animals to *Anopheles gambiae*, *Triatoma infestans* and *Phlebotomus argentipes* saliva were also analyzed to test the efficacy of insecticide-treated nets to protect humans and animals against vector bites [24]–[26]. These studies provided a proof of concept for the application of anti-saliva antibodies as immunological tool for vector control interventions.

The major difficulties in developing an immunological test to detect vector exposure include a) problems in rearing sufficient numbers of the respective arthropod, b) the collection of arthropod saliva, such as from sand flies or mosquitoes and c) technical difficulties in dissecting salivary glands if saliva cannot be obtained [17], [27]. To surmount these difficulties, recombinant immunogenic salivary proteins were developed for immunoassays to replace saliva. These recombinant proteins improved the sensitivity, specificity and reproducibility of the tests. An example is the well-studied 10 kDa salivary protein of *Anopheles gambiae*, a highly conserved protein in the *Anopheles* genus [28]. This protein was expressed recombinantly (gSG6), and characterized for its immunogenicity and especially its *Anopheles*-specificity using sera of children exposed to *A. gambiae* [29], [30]. This protein is an ideal candidate exposure marker for the main African tropical malaria vectors because levels of anti-gSG6 antibodies dropped quickly after the end of the mosquito season [31], [32]. Other examples of successfully characterized recombinant exposure markers are a recombinant calreticulin protein that was evaluated as a promising marker of tick exposure using human sera [33], [34] and two heterologous synthesized salivary proteins of *Lutzomyia longipalpis* that proved to be more effective and sensitive in immunoassays compared to crude sand fly saliva [35], [36].

A recombinant salivary exposure marker, r*Ti*SP14.6, was developed recently to detect exposure to *Triatoma infestans*, the most effective vector of Chagas disease [37]–[39]. R*Ti*SP14.6 was very effective in detecting differences in infestation levels of *T. infestans* in Bolivian households by analyzing IgG levels against the corresponding salivary protein using chicken sera. IgM antibodies of chicken sera also reacted with r*Ti*SP14.6, but compared to IgG immune responses of chickens no differences were detectable in the overall antibody reactions to either crude saliva or r*Ti*SP14.6 comparing chicken sera from lowly or highly *T. infestans* infested households [38]. Guinea pig sera were also tested with r*Ti*SP14.6, but the recombinant antigen only reacted very weakly with guinea pig antibodies in immunoassays, and no differences between *T. infestans* infestation levels were evident using laboratory or field sera [37]. Compared to chickens, mammalian hosts of *T. infestans*, such as guinea pigs and dogs, are of high importance in the epidemiology of Chagas disease, because they are reservoir hosts of *T. cruzi* [40], [41] and thus represent an important risk factor of peridomestic and domestic *T. cruzi* transmission to animals and humans [42], [43]. Thus, the development of an immunological marker that would react with guinea pig sera as a detector of triatomine exposure and a risk marker of *T. cruzi* infection, respectively, would be beneficial for surveillance in *T. infestans* vector control.

Saliva of hematophagous species can differ in its composition between populations of the same species such as analyzed for sand flies [44] and triatomines [37], [45], [46]. Additionally, salivary proteins of arthropods vary not only between populations but also between developmental stages [47], [48]. Such variations in the saliva composition between arthropod populations and between developmental stages may induce different humoral responses in the vertebrate's hosts. In order to develop an appropriate *T. infestans* exposure marker, in particular a salivary antigen that will be recognized by sera of guinea pigs exposed to any developmental stage or strain of *T. infestans*, we characterized the antibody response of guinea pigs exposed to a low number of nymphal or adult *T. infestans*. Experimental animals were exposed to different *T. infestans* strains, originating from Argentina, Bolivia, Chile and Peru.

## Materials and Methods

### Ethics statement

All experimental exposures of animals to triatomines carried out in the Czech Republic were in accordance with the Animal Protection Law of the Czech Republic (§17, Act No. 246/1992 Sb) and with the approval of the Academy of Science of the Czech Republic (protocol approval no. 172/2010) which complies with the regulations of the European Directive 2010/63/EU on the protection of animals used for scientific purposes in Europe. All animal experiments performed in Peru were approved by the Institutional Animal Care and Use Committee (IACUC) of the Universidad Peruana Cayetano Heredia (protocol approval no. 60068). This committee acts in accordance with the US federal laws and thus all animal experiments in Peru followed the guidelines of the US Animal Welfare Act.

### *Triatoma infestans* and saliva collection

The four different strains of *T. infestans* originated from Argentina, Bolivia, Chile and Peru (for further details, see Table S1). Triatomine colonies were reared at an air temperature of 28±1°C, a relative humidity of 60–70% and with a 12 h/12 h light/dark cycle. For colony maintenance the bugs were regularly fed on guinea pigs or rabbits. All experiments were performed with saliva of *T. infestans*, starved for one week after the last blood meal. This period of time after a blood meal is necessary to re-synthesize saliva components injected into the host during the previous feeding [49]. The saliva (0.5–1 µl per bug) was collected typically from either 100 nymphs (5^th^ instar) or 50 adults (females and males) using glass Pasteur pipettes as previously described [37]. The protein concentration was determined using the BCA Protein Assay Kit (Thermo Scientific) according to the manufacturer's instructions and the saliva was and stored at −80°C until use. For the analysis of the developmental stage-specific salivary proteins of *T. infestans* saliva was collected from 20–50 bugs of each instar including males and females separately.

### Exposure experiments

Guinea pigs (four months old) used for exposure experiments in the Czech Republic were obtained from our in-house breeding and animal facility and guinea pigs (four months old) in Peru were purchased from an animal distributor in Lima, Peru and had never been in contact with triatomines. In the laboratories of the Czech Republic and in Peru guinea pigs were exposed weekly to *T. infestans* over a period of 10 weeks to induce a strong anti-triatomine saliva antibody response. Two different experimental procedures were carried out in the Czech Republic and in Peru as described in the following. In the Czech Republic a group of 18 guinea pigs was divided into three subgroups of six animals, and each subgroup was either exposed to a triatomine strain from Argentina or Bolivia or Chile. Within each subgroup, three guinea pigs were either challenged with five 5^th^ instars or five adult *T. infestans*. The triatomines were allowed to probe 50 times for 1 min on the animals during each exposure experiment. In Peru a group of 3 guinea pigs was exposed to a mixture of 5^th^ instars and adults of a Peruvian *T. infestans* strain (10 nymphs and 10 adults) for 1 h. A control group of three guinea pigs was not exposed to triatomines in both laboratories and all guinea pigs in the Czech Republic and Peru were bled from the hind leg from the vena saphena. Pre-exposure sera were taken from all animals as negative controls for immunoassays. Starting at five days after the first exposure to triatomines, blood was sampled in weekly intervals.

### ELISA

IgG and IgM antibodies in guinea pig sera were analyzed by ELISA as previously described using either 0.5 µg protein of crude nymphal (5^th^ instar) or adult saliva/well [37]. Briefly, IgG antibodies were detected using horseradish peroxidase (HRP) conjugated rabbit anti-guinea pig IgG secondary antibodies (Sigma-Aldrich) diluted 1∶20,000 in PBS-Tween20 (PBST) with 2% dried skimmed milk. For the detection of IgM antibodies, HRP goat anti-guinea pig IgM secondary antibodies (Immunology Consultants Laboratory) were used and diluted 1∶10,000 in PBST with 2% dried skimmed milk. The ninety-six well plates (Medisorp, Nunc) were developed using orthophenylenediamine (Sigma-Aldrich) with hydrogen peroxide. The reaction was stopped with 2 M H_2_SO_4_, and optical densities (OD) were measured at 490 nm with a Multiskan MCC/340 spectrophotometer (Labsystems Oy). All samples were analyzed in duplicates and tested in two independent ELISA assays. A sample O.D._490 nm_ was determined by calculating the mean O.D._490 nm_ of the duplicated sample and by subtracting the O.D._490 nm_ of the pre-exposure sample.

### 1D-gel electrophoresis

For the analysis of 1) the salivary protein profiles of all developmental stages of the Bolivian *T. infestans* strain and 2) of 5^th^ instars and adults of the different *T. infestans* strains, crude saliva (1 µg protein/lane) was separated by SDS-PAGE on 15% acrylamide gels under reducing conditions as previously described [37]. Briefly, denatured salivary proteins were separated electrophoretically at 150 V for 50 min following 300 V for 1.5 h. Afterwards acrylamide gels were stained using the SilverQuest staining kit (Invitrogen). Molecular weights were calculated with reference to the mobility of the standard proteins from the SeeBluePlus2 Prestained Standard (Invitrogen) using the software TotalLab TL120 (TotalLab). The level of similarity between the salivary profiles of each developmental stage or between the strains of *T. infestans* was analyzed using the software FreeTree [50]. Based on the presence or absence of salivary bands a distance matrix was created from which the Nei-Li/Dice's coefficient of similarity was calculated and used to construct an UPGMA (Unweighted Pair Group Method with Arithmetic Mean) phenogram. The robustness of trees was assessed by bootstrap analysis (1000 bootstrap replicates). The dendrogram was displayed using TreeView [51].

### Immunoblot

Nymphal (5^th^ instar) or adult salivary proteins (50 µg protein in a 2D well) were separated by SDS-PAGE as described above and transferred onto a nitrocellulose membrane at 25 V for 30 min using the Pierce Fast Semi-dry Blotter (Thermo Scientific). After the transfer, the membrane was blocked with 5% skimmed milk in PBS overnight at 4°C, washed three times in PBST and incubated with guinea pig sera diluted 1∶100 in PBST with 5% skimmed milk for 1 h at room temperature using the Mini-Protean II Multiscreen apparatus (Bio-Rad). Following repeated washing steps, the Western blot membrane was incubated for 1 h at room temperature with HRP conjugated rabbit anti-guinea pig IgG secondary antibodies (Sigma-Aldrich) diluted 1∶10,000 in PBST with 5% dried skimmed milk or HRP goat anti-guinea pig IgM secondary antibodies (Immunology Consultants Laboratory), diluted 1∶5,000 in PBST with 5% dried skimmed milk. The proteins were visualized using the Pierce ECL Western Blotting Substrate (Thermo Scientific) and digitized using the LAS-3000 machine (Fujifilm).

### 2D-gel electrophoresis

Polyacrylamide gel strips with a non-linear pH gradient 3–11 (13 cm, Immobiline DryStrip, GE Healthcare) were incubated in a rehydration buffer (2 M thiourea, 7 M urea, 2% chaps, 15 mM DTT) containing 0.5% carrier ampholytes, pH 3–11 (GE Healthcare) for 12 h at room temperature. Afterwards, desalted crude saliva of 5^th^ instars or adult *T. infestans* (150 µg protein) was applied by cup loading and separated by isoelectric focusing (IEF) using an IEF 100 unit (Hoefer). The saliva was diluted in rehydration buffer and focused at 20°C with a maximum current setting of 200 µA/strip using the following voltage gradient: 0–250 V for 30 min; 250–500 V for 1 h; 500–1000 V for 1 h; 1000–8000 V for 2.5 h; constant 8000 V for 1 h. For the second dimension, the IPG strips were equilibrated in equilibration buffer (6 M urea, 75 mM Tris-HCl, 29.3% (v/v) glycerol, 2% SDS, 65 mM DTT, 0.002% (w/v) bromphenol blue) for 15 min followed by a 15 min incubation with 135 mM iodoacetamide. The strips were placed onto 15% SDS-PAGE gels and the proteins were separated at 300 V and 25 mA for 6 h and stained with silver as described above.

### 2D-western blots

For the Western blot analyses polyacrylamide gel strips with a non-linear pH gradient 3–5.6 (13 cm, Immobiline DryStrip, GE Healthcare) were rehydrated as describe above. Afterwards, 40 µg protein of desalted, nymphal (5^th^ instar) or adult saliva was focused at 20°C with a maximum current setting of 200 µA/strip using cup loading and the following voltage gradient: 0–250 V for 30 min; 250–500 V for 1 h; 500–1000 V for 1 h; 1000–8000 V for 2.5 h; constant 8000 V for 1 h, followed by second dimension as described above but using a transfer time of 1 h. For the Western blot analyses replica gels of the 2D-gel electrophoresis were probed with early sera (5^th^ week) from guinea pigs exposed to nymphal (5^th^ instar) or adult *T. infestans*. Afterwards, Western blot images were compared with the silver stained 2D-gels using program Progenesis SameSpot (Nonlinear dynamics).

### Statistical analysis

The mean O.D._490 nm_ of each subgroup of guinea pigs (3 guinea pigs/subgroup) was calculated for each independent ELISA assay. Therefore, the mean O.D._490 nm_ of the calculated mean sample O.Ds._490 nm_ (mean O.Ds._490 nm_ of duplicated samples and subtraction of the O.D._490 nm_ of the pre-exposure sample) of the three guinea pigs from each subgroup was calculated. This calculation was performed for the O.D. values (subgroups) of the two independent ELISA assays separately. Afterwards, the final mean O.Ds._490 nm_ for all guinea pig subgroups from the two independent ELISA assays was calculated.

ELISA data (final mean O.D._490 nm_ of two ELISA assays) from the long-term exposure study with guinea pigs were statistically analyzed using SPSS, version17.0 (IBM). To compare antibody levels of experimentally exposed guinea pigs, the average from the final mean O.D._490 nm_ (as described above from two independent ELISA assays) of three measured guinea pig sera per each animal subgroup was calculated and the averages of each subgroup were statistically compared. Because the ELISA data did not follow a Gaussian distribution, the non-parametric Wilcoxon signed-rank test was used to compare the antibody levels of guinea pigs tested with only nymphal saliva with the levels of animals analyzed with adult triatomine saliva. The Friedman test was used to uncover differences in the antibody responses between guinea pigs exposed to different *T. infestans* strains. Differences in all tests were considered as statistically significant at a level of p<0.05.

Correlations between the sum of feeding triatomines (number of triatomines used at each feeding event plus the number of triatomines already fed in previous events) per feeding event of each *T. infestans* strain and the corresponding guinea pig IgG antibody levels (mean O.D._490 nm_) were analyzed using the non-parametric Spearman rank correlation test. Because a set of nymphal and adult Peruvian *T. infestans* and a higher number of Peruvian triatomines were used in guinea pig experiments compared to guinea pigs challenged with *T. infestans* from Bolivia, Chile and Argentina, datasets from the Peruvian *T. infestans* exposure experiments were analyzed separately. In all cases, antibody levels either measured against nymphal or adult saliva in ELISA assays were used in separate correlation tests in order to differentiate between developmental specific antibody responses. We further fit parametric models to describe the relationship between the cumulative number of insect bites and the O.D._490 nm_ of each sample taken at each time point using R [52]. We compared simple linear relationships to logistic curves; in all cases the logistic models had lower residual errors than the linear models.

## Results

### Salivary proteins of the developmental stages and strains of *T. infestans*

**Figure 1:**
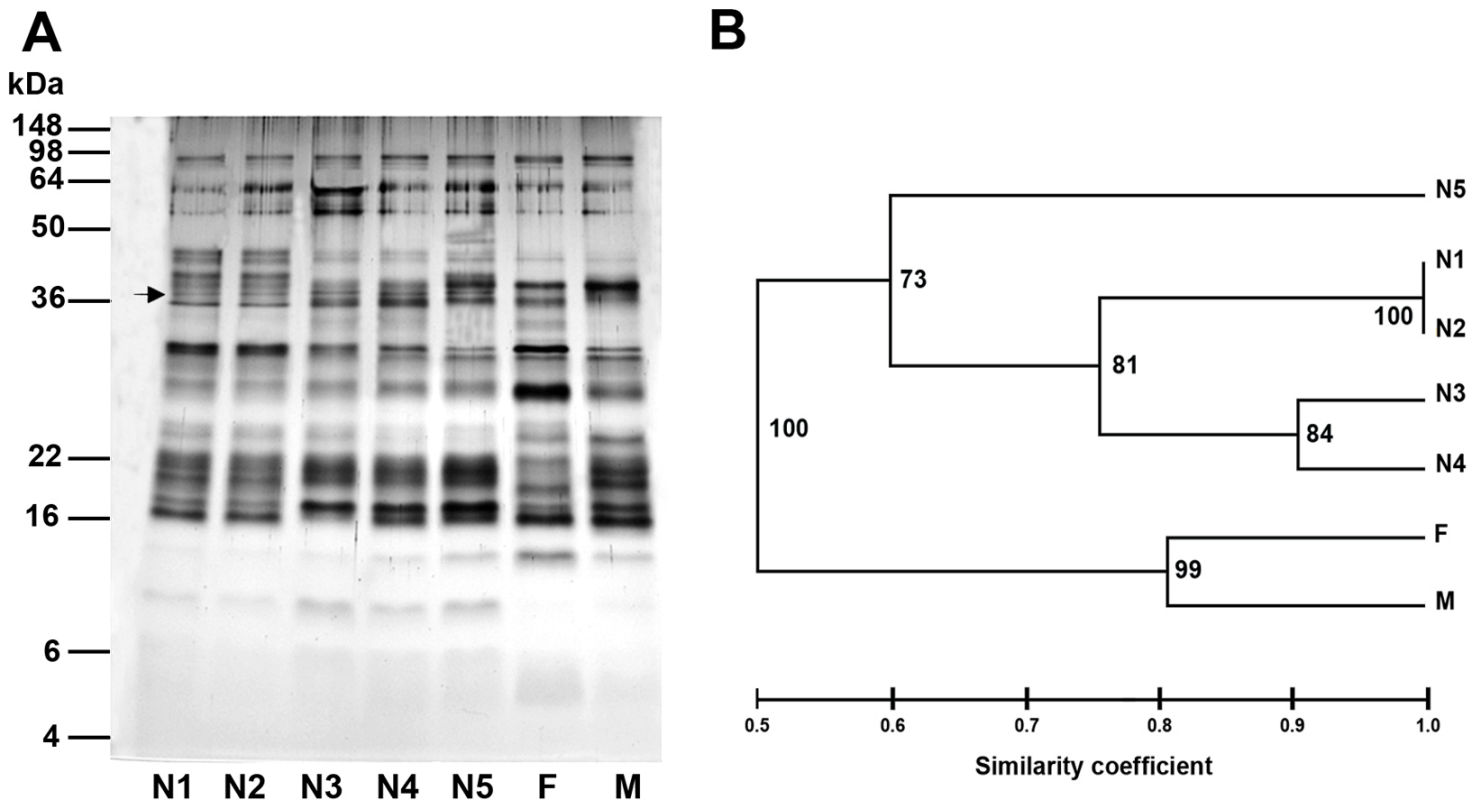
Salivary profiles of the developmental stages of *T. infestans*. (A) Proteins in crude saliva of all nymphal (N1–N5) and adult (female, F and male, M) stages of a Bolivian *T. infestans* strain were separated by SDS-PAGE. A nymphal-specific salivary protein of 37 kDa is marked with an arrow. (B) An unrooted phenogram of the salivary profiles was constructed using the UPGMA method of the FreeTree software [50]. Bootstrap values are displayed at the nodes of the tree and the scale bar represents the Dice's similarity coefficient.

Analysis of the saliva content of all developmental stages of *T. infestans* (pooled saliva of 20–50 bugs per developmental stage) revealed a complex pattern of salivary proteins ranging from 5 kDa to 92 kDa as demonstrated for the Bolivian *T. infestans* strain (Figure 1A). Most differences in the salivary profiles were detected between nymphs and adults, e.g. a protein band of 37 kDa was present in the saliva of all nymphal stages but not in adult saliva (Figure 1A, marked with an arrow). An unrooted phenogram constructed from the electrophoretic saliva profiles of the different developmental stages disclosed two main groups that separate nymphal salivary proteins from adult proteins with the Dice's similarity coefficient of 0.5 (Figure 1B). The first two nymphal instars (N1 and N2) shared the same protein bands (similarity coefficient = 1.0), and between the following nymphal instars the degree of similarity decreased. Thus, the salivary profile of the 5^th^ instar was the most different compared to all other nymphal profiles (similarity coefficient = 0.60). The salivary protein pattern of female and male *T. infestans* were quite similar (similarity coefficient = 0.80).

**Figure 2:**
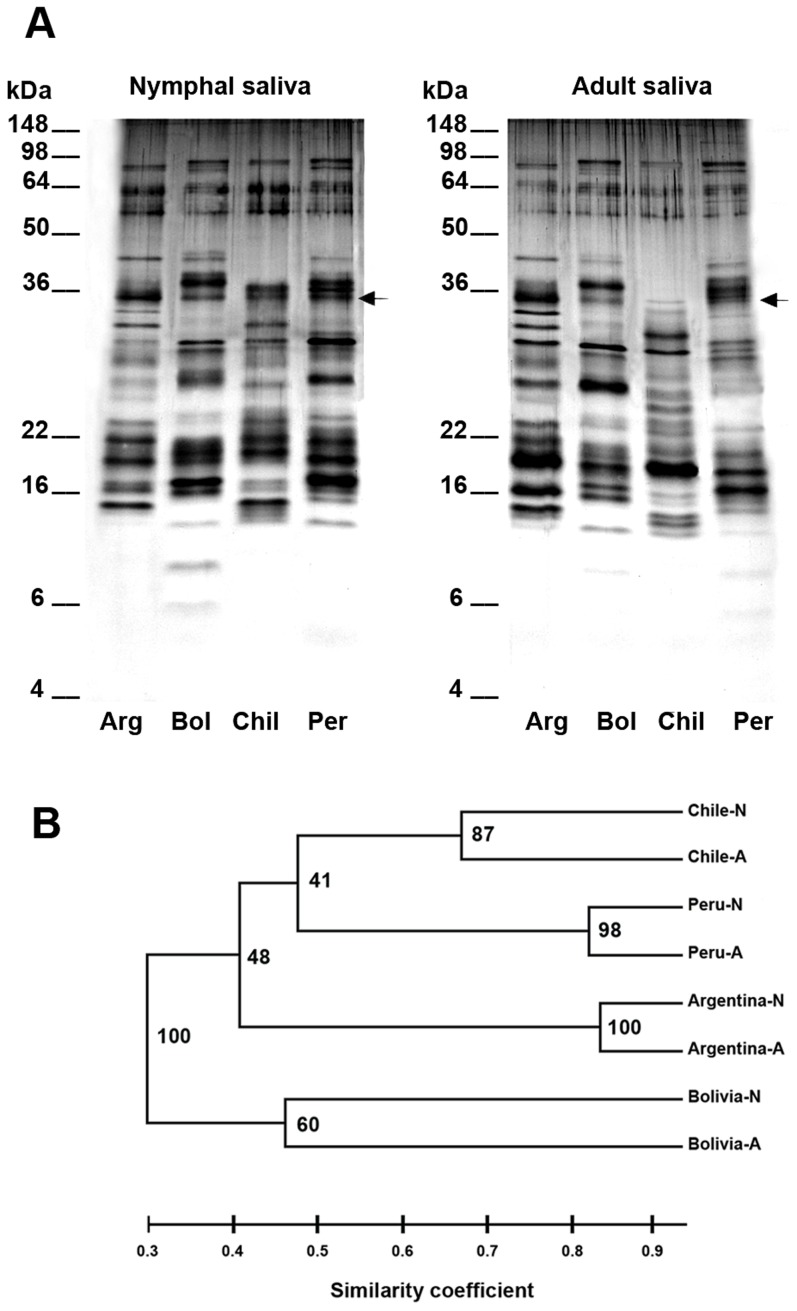
Nymphal and adult salivary profiles of four different *T. infestans* strains. (A) Saliva of starved nymphs (fifth instar) and adults (pooled saliva of females and males) of four different *T. infestans* strains from Argentina (Arg), Bolivia (Bol), Chile (Chil) and Peru (Per) were analyzed by SDS PAGE. Arrows mark the protein band of 35 kDa that is common for all *T. infestans* strains. (B) An unrooted phenogram was constructed from the electrophoretic salivary profiles of nymphs (N) and adults (A) of the different *T. infestans* strains using the UPGMA method of the FreeTree software [50]. Bootstrap values are displayed at the tree nodes and the scale bar represents the Dice's similarity coefficient.

Due to the phenogram's analysis saliva of the 5^th^ instar and adults of each *T. infestans* strain were separated by gel electrophoresis in order to compare the saliva composition of different *T. infestans* strains (Figure 2A). Salivary protein profiles of the different *T. infestans* strains showed less similarity (similarity coefficient = 0.3) compared to profiles of nymphs and adults of each strain (similarity coefficient = 0.46–0.84, Figure 2B). Comparing all salivary protein profiles, nymphal and adult profiles of the Bolivian *T. infestans* strain were most different to the profiles of all other strains (similarity coefficient = 0.3, Figure 2B). Despite these variations in the saliva composition, several protein bands were shared between the different *T. infestans* strains such as of the 25 kDa, 30 kDa, 33 kDa, 35 kDa (Figure 2A, marked with arrows), 55 kDa and 62 kDa proteins.

### Antibody responses of guinea pigs to salivary antigens of *T. infestans*

Because most differences in the salivary profiles were detected between the 5^th^ nymphal *T. infestans* stage and adult triatomines, both stages were used to induce antibody responses in guinea pigs. To characterize the development of IgG and IgM antibody responses guinea pigs were either bitten a) by bugs of the 5^th^ instar or adults from the Argentinean, Bolivian or Chilean *T. infestans* strain or b) by a set of nymphs and adults of the Peruvian strain. All sera of guinea pigs (including sera from guinea pigs exposed to Peruvian triatomines) were either analyzed with saliva of the 5^th^ instar or adults of the different *T. infestans* strains.

**Figure 3:**
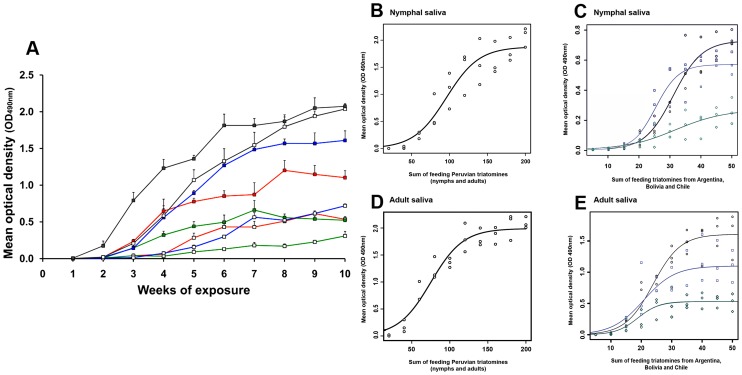
IgG antibody response of guinea pigs to saliva of nymphal and adult *T. infestans* of four different strains. (A) Eighteen guinea pigs were either exposed to the 5^th^ instar (n = 5, white squares) or adults (n = 5, colored squares) of three different *T. infestans* strains from Argentina (blue line), Bolivia (red line) and Chile (green line). Each group of animals was made of 3 guinea pigs. Additionally, three guinea pigs were exposed to a set of nymphs (n = 10, white squares) and adult triatomines (n = 10, grey squares) from Peru (grey lines). Guinea pigs were exposed weekly to triatomines and for a period of 10 weeks. Animals were bled 5 days after each exposure and all sera were analyzed by ELISA using either crude saliva of nymphs or adults. From each group of guinea pig sera (n = 3) the mean optical density (O.D._490 nm_) was calculated after subtracting the O.D. of the negative control (pre-exposure). The results here presented show the final mean optical densities (O.D._490 nm_) of two independent ELISA assays. (B–E) Logistic models describing the relationship between the sum of feeding nymphal and/or adult triatomines (number of triatomines used at each feeding event plus the number of triatomines already fed in previous events) and the corresponding IgG antibody level of guinea pigs to saliva of the Peruvian (B, D), Argentinean (C, E, black graphs), Bolivian (C, E, blue graphs) and Chilean *T. infestans* strains (C, E, green graphs).

The level of IgG antibodies increased in guinea pigs bitten by nymphal and/or adult triatomines from Argentina, Bolivia, Chile and Peru with serial exposures to *T. infestans* (Figure 3A). Anti-saliva IgG antibodies of guinea pigs exposed to the Peruvian *T. infestans* strain were detectable after the first exposure, although at a very low level (mean O.D. _490 nm_ = 0.003, Figure 3A). All other IgG antibody levels were detectable after the second exposure, at which some antibody levels were also very low (mean O.D. _490 nm_ range = 0.003–0.016). The highest antibody levels were reached in guinea pigs exposed to the Peruvian strain (mean max. O.D. _490 nm_ = 2.073).

Anti-saliva IgG levels of guinea pigs challenged with only nymphs of either the Argentinean, the Bolivian or the Chilean *T. infestans* strain increased significantly slower (max. mean O.D._490 nm_ range = 0.238–0.766) compared to the antibody levels of guinea pigs exposed to adults only (max. mean O.D._490 nm_ range = 0.574–1.669; Wilcoxon signed-rank test, p<0.001); these guinea pigs reached lower antibody levels in general. Although some guinea pigs were exposed to a set of nymphal and adult triatomines from Peru, significant differences in the IgG antibody response were detected when using either only nymphal or adult Peruvian saliva in ELISA assays (Wilcoxon signed-rank test, p<0.001). Moreover, IgG antibody responses of animals exposed to adult triatomines of all strains differed significantly (Friedman test, p<0.001, Figure 3A) but no significant differences appeared between the immune response of guinea pigs exposed to nymphs of the different *T. infestans* strains (Friedman test, p>0.05). Overall, the sum of feeding nymphal and/or adult triatomines on guinea pigs (number of triatomines used at each feeding event plus the number of triatomines already fed in previous events) of all *T. infestans* strains correlated significantly and strongly, positively with the IgG antibody level of guinea pigs over the experimental period of triatomine exposure (for statistics please see Table S3). But a linear relationship between the number of biting triatomines and the corresponding IgG antibody response was only measurable until the 7^th^ or 8^th^ week of triatomine exposure (35–40 fed bugs) when using the *T. infestans* strains from Argentina, Bolivia and Chile (Figure 3C and E) and until the 6^th^ or 7^th^ week of triatomine exposure (120–140 fed bugs) when using Peruvian *T. infestans* (Figure 3B and D). Afterwards, the relationship between the IgG antibody response and the number of biting triatomines saturated.

**Figure 4:**
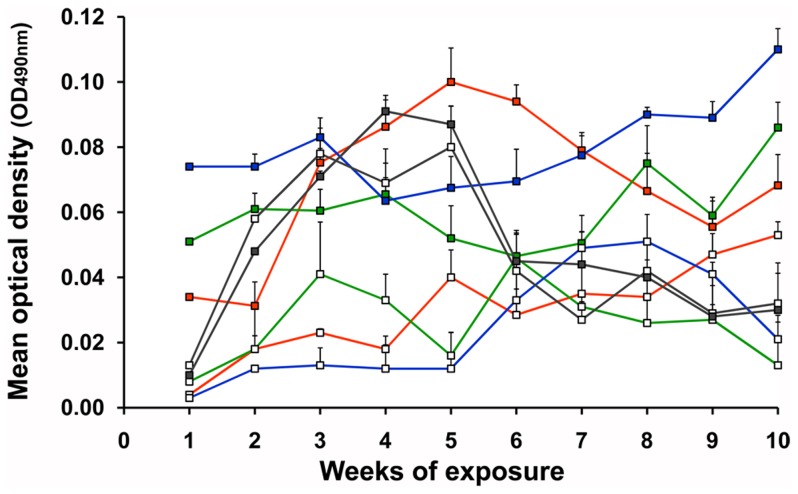
IgM antibody response of guinea pigs to saliva of *T. infestans*. Eighteen guinea pigs were either exposed to nymphs (white squares) or adults (colored squares) of three different *T. infestans* from Argentina (blue line), Bolivia (red line) and Chile (green line). A second group of three guinea pigs was exposed to a set of nymphal (n = 10, white squares) and adult (n = 10, colored squares) *T. infestans* from Peru (grey line). Sera from all exposure events were tested either with crude saliva of nymphs or adults in ELISA assays to monitor the development of the IgM antibody response in guinea pigs. Mean optical densities (O.D._490 nm_) of each exposure subgroup (3 guinea pig sera) were calculated after subtracting the O.D.s of the negative controls (pre-exposures). The final mean O.D.s presented in this graph were calculated from two independent ELISA assays.

IgM antibodies of all guinea pigs to crude saliva of nymphal (5^th^ instar) and/or adult *T. infestans* were detectable after the first exposure to triatomines (max. mean O.D._490 nm_ = 0.074, Figure 4). Compared to IgG antibody responses (Figure 3A), IgM responses of guinea pigs to triatomine saliva were very low and they only fluctuated slightly during the serial exposure to triatomine bites (max. mean O.D._490 nm_ range = 0.004–0.110). No significant differences between the IgM responses were detected using either nymphs and/or adults of all strains in the exposure study (Wilcoxon signed-rank test, p>0.05, Figure 4), and the overall sum of feeding nymphal and/or adult triatomines of all strains were not correlated with IgM antibody levels (Spearman rank correlation test, p>0.05).

### Immunogenic salivary antigens of *T. infestans*

**Figure 5:**
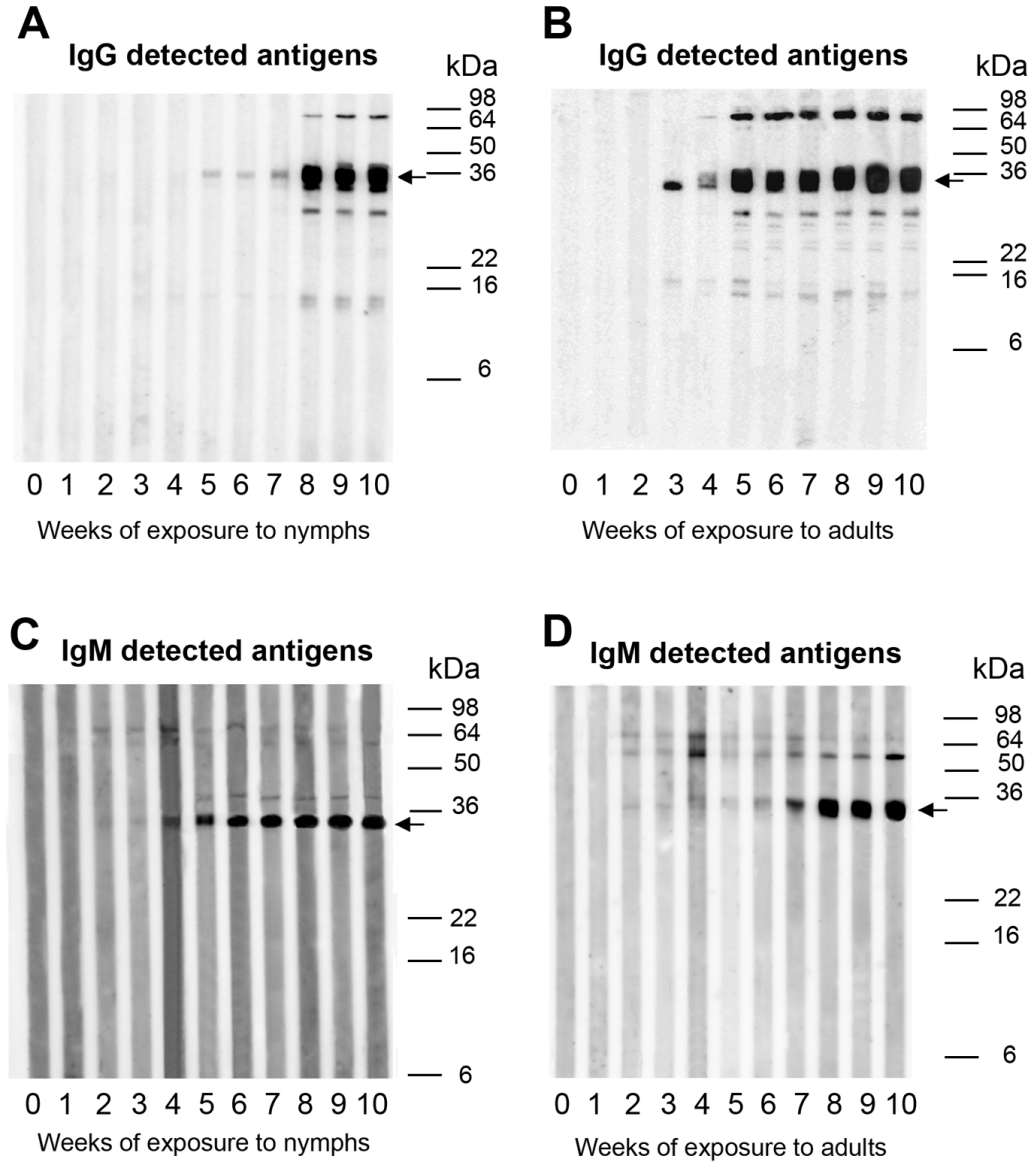
Immunogenic salivary antigens of *T. infestans*. Sera of guinea pigs that were weekly exposed to nymphal (A, C) or adult *T. infestans* (B, D) of the Bolivian strain over a period of 10 weeks (1–10) were used in Western blot experiments to detect IgG (A, B) and IgM (C, D) reacting antigens. A serum from a guinea pig prior to the exposure to bug bites was used as a negative control (0) in the Western blot experiments to demonstrate the specificity of the IgG and IgM anti-saliva *T. infestans* responses. Arrows mark the 35 kDa salivary protein that was detected in all Western blots by IgG and IgM antibodies.

Guinea pig sera were used to detect immunogenic salivary proteins. Figure 5 represents an example of developmental stage-specific antigens of the Bolivian *T. infestans* strain. Antigen reactions of all four strains are summarized in Table S2. IgG reacting antigens were mainly detected from the third week of triatomine exposure (Figure 5A and B), but some bands were already visible on Western blots from the second week of triatomine exposure using sera of guinea pigs exposed to the Peruvian strain (data not shown). More salivary proteins of adults than of 5^th^ instar nymphs were recognized by IgG antibodies (Figures 5A and B, Table S2). In contrast to IgG reactions, IgM antibodies bound to salivary proteins already after the first or second week of triatomine exposure (Figure 5C and D). Only a few proteins were detected in nymphal and adult saliva using IgM antibodies, and no antigens below 30 kDa of the Bolivian *T. infestans* strain reacted with IgM antibodies.

Strain-specific variations in the recognition of salivary proteins by antibodies were also evident in Western blots (Table S2). Using IgG secondary antibodies, most antigens were detected for the Chilean strain (nymphal antigens = 12, adult antigens = 15) while only 8 proteins of adult saliva from Argentinean triatomines were immunogenic (Table S2). IgM reactions with saliva of the Argentinean, Chilean and Peruvian *T. infestans* strains were similar to the reactions with saliva of the Bolivian strain; less proteins reacted with IgM antibodies but a few proteins with molecular weights <30 kDa were detected. The differences observed in the antibody response of the Peruvian *T. infestans* strain when using only nymphal or adult saliva in ELISA assays were also reflected in the Western blots; IgG or IgM antibodies did not bound entirely to the same salivary proteins using either nymphal or adult saliva. Despite the differences in developmental-stage and -strain specific antigens of *T. infestans*, a 35 kDa antigen was detected by sera of almost all guinea pigs using both IgG (19 out of 21) and IgM (18 out of 21) antibodies (Table S2).

**Figure 6:**
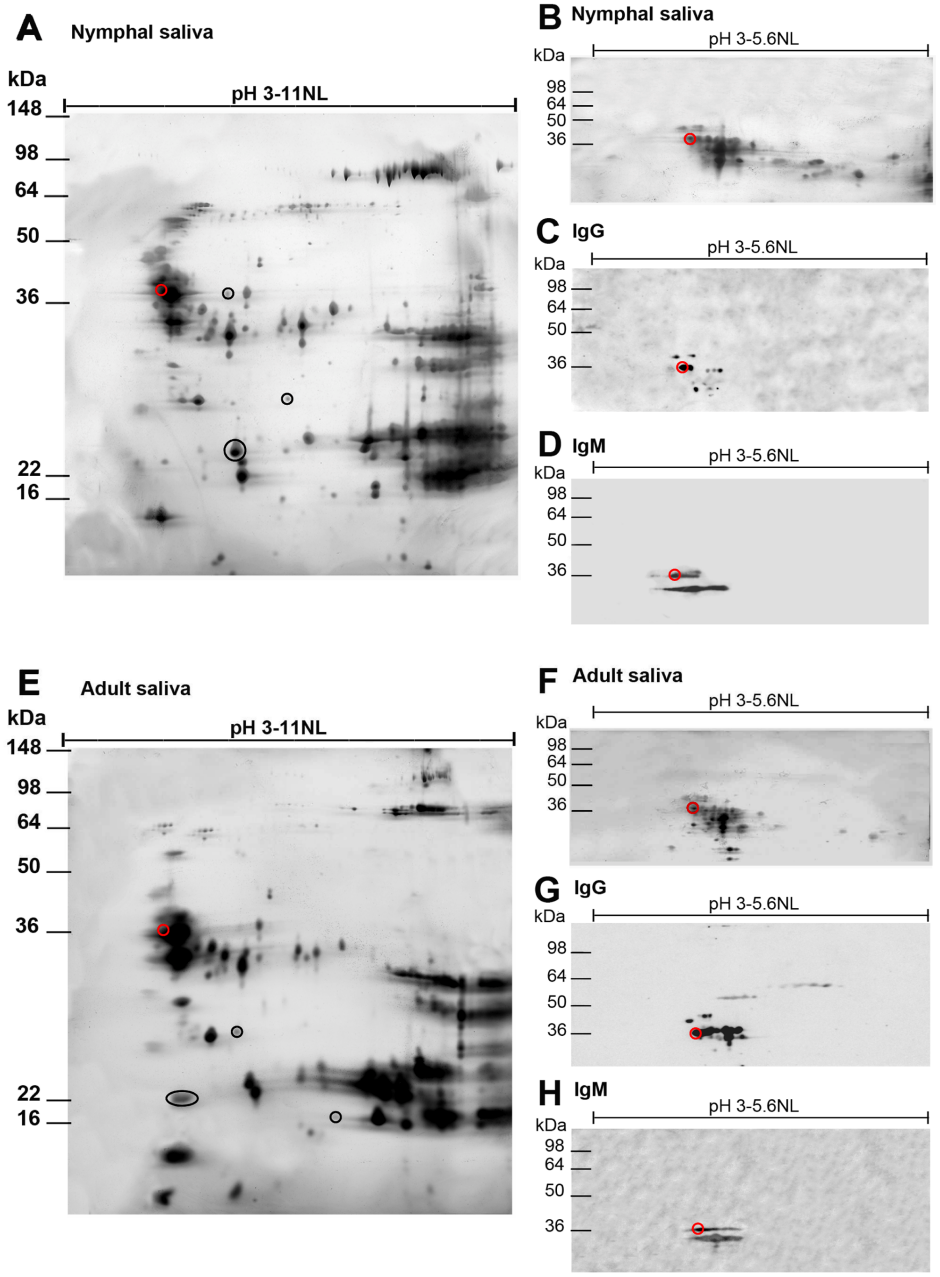
Characterization of the 35*T. infestans*. Salivary proteins of *T. infestans* were analyzed by 2D gel electrophoresis and 2D Western blotting in order to identify the candidate exposure marker protein of 35 kDa. The figure presents an overview of nymphal (5^th^ instar, A) and adult (females and males, E) salivary proteins of the Bolivian *T. infestans*. Developmental stage specific salivary proteins in nymphal (A) and adult (E) saliva are marked with black circles. In order to improve the protein separation, crude saliva of nymphs (B) and adults (F) were isoelectric focused in the nonlinear pH range of 3–5.6. Focused proteins were blotted onto nitrocellulose and tested for their immunogenicity using a guinea pig serum from the 5^th^ week of triatomine exposure. IgG (C, G) and IgM antibody reactions (D, H) with nymphal (C, D) and adult (G, H) *T. infestans* salivary proteins were analyzed. The candidate exposure marker antigen of 35 kDa detected by IgG and IgM antibodies is marked with a red circle.

In order to further characterize the 35 kDa antigen of *T. infestans* that may be a potential triatomine exposure marker, proteins were separated by 2D SDS PAGE, using a nonlinear pH range of 3–11 (Figures 6A, E and Figures S1, S2, S3A, E), and used for Western blots. In accordance with Figure 5, Figure 6 represents an example of 2D salivary protein patterns and Western blots of saliva of 5^th^ instar nymphs (A–D) and adults (E–H) of *T. infestans* from Bolivia. The 2D salivary protein profiles of the Chilean, Argentinean and Peruvian *T. infestans* strain are presented in the Figures S1, S2, S3. Although *T. infestans* proteins appeared almost along the entire pH range, concentrations of proteins were focused at pH 3.8–4.6 and 8.4–11.0. 2D gel analyses revealed much more precisely and detailed the differences in the salivary profile between the 5^th^ instars and adults of *T. infestans* as shown in the Figures 6A and E and Figures S1, S2, S3A and E (some examples of nymphal- or adult-specific protein spots are marked).

Experimental guinea pig sera from the fifth week of exposure were used to detect the candidate exposure marker antigen of 35 kDa. Since this antigen had a pH of 4.1 and thus accumulated in the 2D gels with other *T. infestans* proteins of the same pH, salivary proteins of nymphs and adults were isoelectrically focused at a pH range of 3–5.6 for a better separation of the salivary proteins (Figures 6B, F and Figures S1, S2, S3B, F) and blotted onto nitrocellulose. A maximum of 9 and 12 salivary protein spots of about 35 kDa in nymphal and adult saliva of the Bolivian *T. infestans* strain, respectively, were recognized by IgG antibodies (Figure 6C, G). In contrast, IgM antibodies reacted with less protein spots of 35 kDa; a maximum of 4 spots from nymphal saliva of the Bolivian *T. infestans* strain appeared to be immunogenic and 6 salivary protein spots were recognized by IgM antibodies in adult saliva (Figure 6D, H). Less salivary proteins of 35 kDa from the saliva of the other three *T. infestans* strains were immunogenic (Figures S1, S2, S3C, G). A maximum of 4 protein spots were detected using IgG antibodies, while only maximal 2 spots were detectable with IgM antibodies (Figure S1, S2, S3, D, H) using sera of guinea pigs exposed to nymphs or adults of other strains of *T. infestans*. Of all salivary proteins detected by IgG and IgM antibodies in 2D gel analyses, only one salivary protein of about 35 kDa reacted with both types of immunoglobulins using sera of guinea pigs exposed to nymphs and/or adults of all *T. infestans* strains.

## Discussion

Multinational Chagas disease control initiatives in Latin American countries have greatly reduced the transmission of *T. cruzi* since their establishment in the 1990s [53], [54]. Success in Chagas disease control depends mainly on the elimination of triatomines, especially *T. infestans* [55]–[57]. Although insecticide-based vector control has been very effective and reduced infestation rates throughout Latin America, such as in Chile where domestic populations of *T. infestans* were reduced by 96% between 1982 and 1998 [58], the resistance of *T. infestans* to insecticides [59], [60] and the low effectiveness of insecticides in peridomestic environments, has become a wide-spread problem [61], [62]. Moreover, after successful elimination of triatomine populations conspecific triatomine species can re-infest triatomine-free houses [63], [64]. To detect re-infesting triatomines artificial shelter units or biosensor boxes have been already used, but they are of limited sensitivity for the detection of triatomines [65]–[67]. Thus, highly sensitive surveillance methods are required that can especially detect low level re-infestations of triatomines [55]. A promising epidemiological tool to detect such levels is the use of anti-triatomine saliva antibodies from triatomine hosts that react with salivary antigens of these bugs [37]–[39]. These salivary antigens may be useful markers of triatomine exposure. In the present study we analyzed IgG and IgM antibody responses of guinea pigs that are typical peridomestic hosts of triatomine bugs, to the saliva of different developmental stages and strains of *T. infestans* in order to detect candidate *T. infestans* exposure markers.

Several studies focused on the characterization of immunogenic salivary antigens as potential markers of exposure to detect hematophagous arthropods using sera of different host species [e.g. 14,16,21,37]. Depending on the host species different salivary antigens of *T. infestans* were detected as immunogenic, e.g. 12–59 kDa proteins were recognized by chicken sera, 14–120 kDa proteins by mice sera and 13–81 kDa as well as 8–105 kDa antigens were recognized by sera of Chagas disease patients and non-Chagas disease patients living in triatomine infested areas, respectively [37], [68], [69]. However, in all these studies developmental stage and/or strain-specific differences in salivary proteins were not examined. Our results here emphasize the importance of these covariates on the humoral immune response of the vertebrate hosts. The immunogenicity of salivary proteins differed between developmental stages of *T. infestans* as especially demonstrated for the Peruvian 5^th^ instars and adults. Despite the variability of recognized proteins from the developmental stages and strains of *T. infestans*, a salivary antigen of 35 kDa could be identified as candidate exposure marker using guinea pig sera that was shared by all developmental stages and strains of *T. infestans* using both IgG and IgM antibodies.

Previously, r*Ti*SP14.6, a 14.6. kDa recombinant salivary protein of *T. infestans*, was evaluated as a suitable exposure marker to detect low-level infestation of *T. infestans* using chicken sera [37]–[39]. An immunogenic salivary protein of about 14 kDa also reacted in Western blot experiments with sera of guinea pigs exposed to *T. infestans*, but r*Ti*SP14.6 was not sufficient in reacting with guinea pig sera in immunoassays [39]. Although in previous studies the same immunogenic protein bands were detected in Western blot experiments using sera of exposure experiments with different host species [37], [68], [69], it does not automatically mean that the actual same proteins were detected. Thus, triatomine exposure markers developed for a certain host species may not be applicable for another species and they need to be tested and evaluated individually with sera of the candidate host species. Therefore, our study focused on the humoral immune response of guinea pigs that are important in the epidemiology of Chagas disease. Guinea pigs are usually kept for consumption and trade in different South American countries such as in Peru [70], and they are sometimes maintained inside human dwellings which increases the risk of triatomine exposure and Chagas disease transmission, respectively [43]. Infestation levels of *T. infestans* can be very high in guinea pig enclosures compared to other animal enclosures, and the presence of animals inside of households during the night can increase the vector abundance about 5-fold [43]. Furthermore, high densities of guinea pigs may allow the re-emergence of *T. cruzi* after vector control interventions as assumed in an entomological study of a peri-rural area, southwest of Arequipa, in Peru, where 27% infested *T. infestans* households contained *T. cruzi*-positive triatomines, but no new parasite infections were detected in humans [71]. Intervention strategies to reduce *T. cruzi* transmission rate in animals, in turn, were already proposed and/or applied, including the keeping of animals outside of the houses at night [72], the prevention of triatomine contact with animals by using material for animal enclosures that do not provide hideouts for triatomines [43], [72], [73] or the use of impregnated nets that cover animal enclosures [25], [74]. The efficiency of these interventions needs to be evaluated. Recently anti-triatomine saliva immunoassays were used to examine if guinea pig enclosures with impregnated nets protected the animals against triatomine bites by measuring the antibody level of the guinea pigs against crude saliva of *T. infestans* [25]. However, the study could not completely rule out that cross reacting IgG antibodies to orthologous salivary proteins of other hematophagous arthropods were also detected in the assays when using crude triatomine saliva. Thus, the application of a recombinant salivary antigen of *T. infestans* as a marker of exposure instead of crude saliva will increase the specificity and sensitivity of such immunological tests for Chagas disease intervention measurements. The usefulness of exposure markers in insecticide treated netting studies was demonstrated in the application of the recombinant *A. gambiae* salivary antigen gSG6-P1 to evaluate the efficacy of insecticide treated nets (ITNs) in a malaria-endemic area using human sera [24]. The authors verified the better specificity and sensitivity of gSG6-P1 compared to crude mosquito saliva in immunoassays when comparing human anti-gSG6-P1 and human anti-saliva *A. gambiae* antibody reactions before and after the introduction of ITNs.

A triatomine exposure marker could be also very useful in epidemiologic surveys to complete entomological data in areas where *T. cruzi* transmission is low, especially after vector control measures against triatomines, and the risk of parasite transmission is newly assessed. Vector control in Chagas disease surveillance relies on regularly inspections of sprayed households and the participation of the local communities in notifying about the presence of bugs [75]. However, sustained surveillance measures are often not implemented in endemic Chagas disease countries. High-throughput blood sampling of humans and/or animals on a regular basis to measure not only *T. cruzi* prevalences but also exposure to bug bites may improve Chagas disease control in detecting especially newly establishing triatomine infestation and parasite transmission foci. Nevertheless, a triatomine exposure marker would be not suitable for correlating *T. cruzi* prevalences and vector bites given the complexity of the relationship between vector exposure and parasite infection as occurring, for example, in Arequipa, Peru. Although the majority of the inhabitants and animals are frequently exposed to *T. infestans* in Arequipa, *T. cruzi* infection is very clustered and sparse throughout the city [76], [77].

For vector control purposes, a sensitive exposure marker should be useable shortly after insecticide sprayings to promptly detect re-establishing triatomine populations. Analyses of the IgG antibody responses in guinea pigs revealed a strong correlation between the antibody response and the number of biting triatomines. Higher numbers of bugs elicit a higher antibody response in the triatomine host which was reflected in the antibody responses of guinea pigs exposed to the Peruvian *T. infestans* strain compared to other *T. infestans* strains. However, due to the saturation effect of the IgG antibody response after a couple of exposure events it is possible to distinguish between a low and high antibody response and thus to distinguish indirectly between highly and lowly infested triatomine sites but a concrete determination of triatomine numbers that fed on triatomine hosts is not possible. Furthermore, anti-triatomine saliva IgG antibodies persist in guinea pigs and chickens up to five months after the last exposure to bugs [37]. Thus, IgG reacting exposure markers are not applicable in areas where control measures were recently carried out. Instead, IgM reacting antigens may be an alternative because IgM antibody responses are detectable only a few weeks after the last exposure to bug bites [38]. The use of IgM antibodies instead of IgG antibodies as immunological tool is generally not considered in studies that characterize the antibody responses of hosts to salivary proteins of hematophagous arthropods for the development of an exposure marker.

Even though recombinant salivary proteins increase the specificity and sensitivity in immunoassays compared to saliva, antibodies that cross react with salivary proteins of different hematophagous arthropods will still counteract the specificity of immunological tests. This cross reactivity can be minimized if species specific salivary proteins are selected as candidate exposure markers instead of highly conserved salivary proteins across the hematophagous arthropod genera which can be verified by sequence similarity analysis using public genetic sequence databases such as GenBank [36]. However, candidate exposure markers should be tested experimentally with sera of hosts that were challenged with the bites of hematophagous arthropods other than the candidate arthropod species to verify certainly the specificity of the antigen [36], [39] if the occurrence of other hematophagous arthropods cannot be ruled out [35]. An alternative approach to minimize cross reactivity may be the use of peptides instead of an entire recombinant protein. Several peptides deriving from the recombinant *Anopheles* exposure marker gSG6 were synthetized and one out of five peptides (gSG6-P1) were proven to detect reliably *Anopheles* exposure [29]. In the latter approach cross reactivity was ruled out by similarity searches of the peptides using GenBank, but peptide specificity was not experimentally verified.

Cross reacting antibodies may be, however, a useful tool if they are elicited by salivary proteins of different triatomine species. Thus, an exposure marker would not be only useful for the detection of a single triatomine species but could be used as a universal marker for the detection of different triatomine species. We tested in Western blots if the 35 kDa candidate marker protein of *T. infestans* is recognized by IgG antibodies of guinea pigs exposed to *Triatoma dimidiata*, *Triatoma brasiliensis*, *Triatoma sordida*, *Triatoma vitticeps*, *Panstrongylus megistus* and *Rhodnius prolixus*. Our analysis revealed that the candidate marker protein is recognized by sera of guinea pigs exposed to 3 out of 6 triatomine species (*T. dimidiata*, *T. brasiliensis*, *T. sordida*, data not shown). However, its final reactivity and sensitivity need to be confirmed after the candidate salivary protein of *T. infestans* is identified molecularly, heterologously expressed and the recombinant candidate protein retested with guinea pig sera.

In summary, salivary antigens are increasingly considered as an epidemiological tool to measure the exposure to hematophagous arthropods, however, developmental stage- and strain-specific variations of in the antibody responses of their hosts are often neglected in the development of an exposure marker. Our study revealed a strong variability not only in the salivary protein profiles between the different *T. infestans* strains but also among the different developmental stages. This variability was reflected in the varying humoral immune response of guinea pigs and resulted in the detection of different salivary antigens depending on the developmental stage and strain of *T. infestans*. Despite the variability of salivary antigens, an antigen of 35 kDa reacted with almost all guinea pig sera using both IgG and IgM secondary antibodies in immunoassays. This protein may be a useful marker of *T. infestans* exposure marker in monitoring programs of Chagas disease control campaigns.

## Supporting Information

Figure S1 **2D salivary profiles and 2D western blot analyses of nymphal and adult** ***T. infestans*** **from Argentina.** Saliva of 5^th^ instar nymphs (A) and adults (females and males, E) of Argentinean *T. infestans* were isoelectrically focused in the nonlinear pH range of 3–11. Black circles indicate nymphal (A) or adult (E) specific salivary proteins. In order to improve the protein separation, saliva of nymphs (B) and adults (F) were isoelectrically focused in the nonlinear pH range of 3–5.6 and blotted onto nitrocellulose. Comparing IgG (C, G) and IgM antibody reactions (D, H) with salivary proteins of nymphs (C, D) and adults (G, H), the candidate exposure marker antigen of 35 kDa was recognized by IgG and IgM antibodies of guinea pig serum from the 5^th^ week of exposure to the Argentinean *T. infestans* strain. This protein is marked with a red circle in the different panels.
(PDF)

Figure S2 **2D salivary profile and western blot analyses of nymphal and adult** ***T. infestans*** **from Chile.** Saliva of 5^th^ instar nymphs (5^th^ instar, A) and adults (females and males, E) of Chilean *T. infestans* were isoelectrically focused in the nonlinear pH range of 3–11. Black circles indicate nymphal (A) or adult (E) specific salivary proteins. In order to improve the protein separation, saliva of nymphs (B) and adults (F) were isoelectrically focused in the nonlinear pH range of 3–5.6 and blotted onto nitrocellulose. Comparing IgG (C, G) and IgM antibody reactions (D, H) with salivary proteins of nymphs (C, D) and adults (G, H) *T. infestans* saliva, the candidate exposure marker antigen of 35 kDa was recognized by IgG and IgM antibodies of guinea pig serum from the 5^th^ week of exposure to the Chilean *T. infestans* strain. This protein is marked with a red circle in the different panels.
(PDF)

Figure S3 **2D salivary profile and western blot analyses of nymphal and adult** ***T. infestans*** **from Peru.** Saliva of 5^th^ instar nymphs (5^th^ instar, A) and adults (females and males, E) Peruvian *T. infestans* were isoelectrically focused in the nonlinear pH range of 3–11. Black circles indicate nymphal (A) or adult (E) specific salivary proteins. In order to improve the protein separation, saliva of nymphs (B) and adults (F) were isoelectrically focused in the nonlinear pH range of 3–5.6 and blotted onto nitrocellulose. Comparing IgG (C, G) and IgM antibody reactions (D, H) with salivary proteins of nymphs (C, D) and adults (G, H) *T. infestans* saliva, the candidate exposure marker antigen of 35 kDa was recognized by IgG and IgM antibodies of guinea pig serum from the 5^th^ week of exposure to the Peruvian *T. infestans* strain. This protein is marked with a red circle in the different panels.
(PDF)

Table S1 **Origin of different** ***Triatoma infestans*** **strains.**
(PDF)

Table S2 **Immunogenic salivary antigens of four different** ***T. infestans*** **strains.**
(PDF)

Table S3 **Correlation between sum of feeding** ***T. infestans*** **and IgG antibody response of guinea pigs.**
(PDF)
