# Supplementary figures and images for: Characterization of Guinea Pig Antibody Responses to Salivary Proteins of Triatoma infestans for the Development of a Triatomine Exposure Marker
Source: PLoS Negl Trop Dis. 2014 Apr 3;8(4):e2783. doi: 10.1371/journal.pntd.0002783 (PMC3974673; doi:10.1371/journal.pntd.0002783)

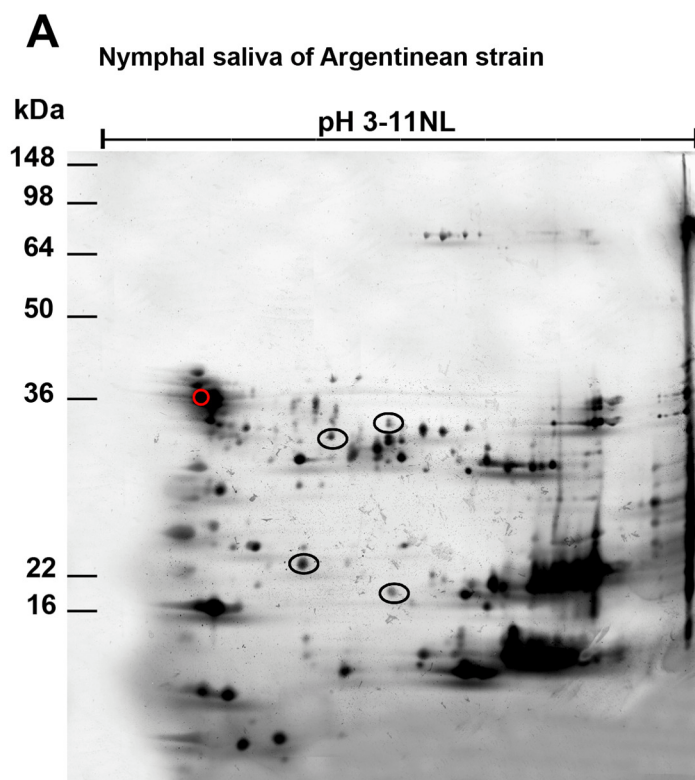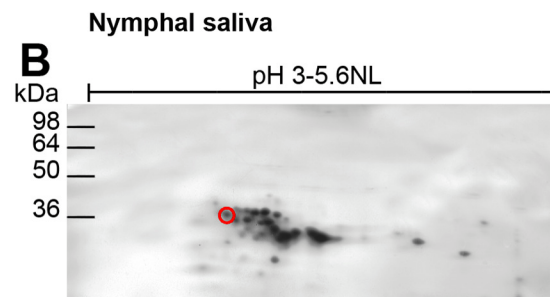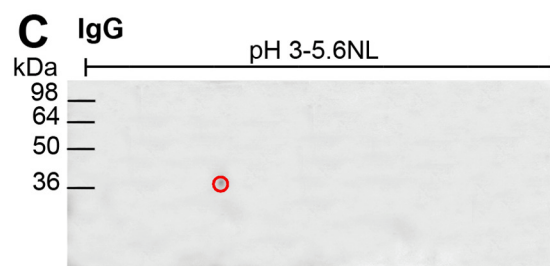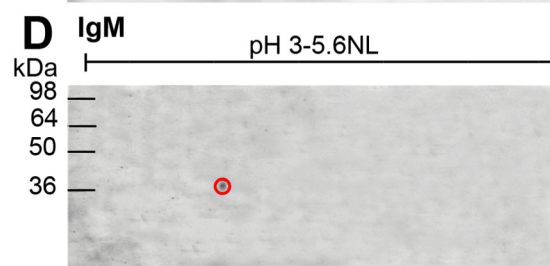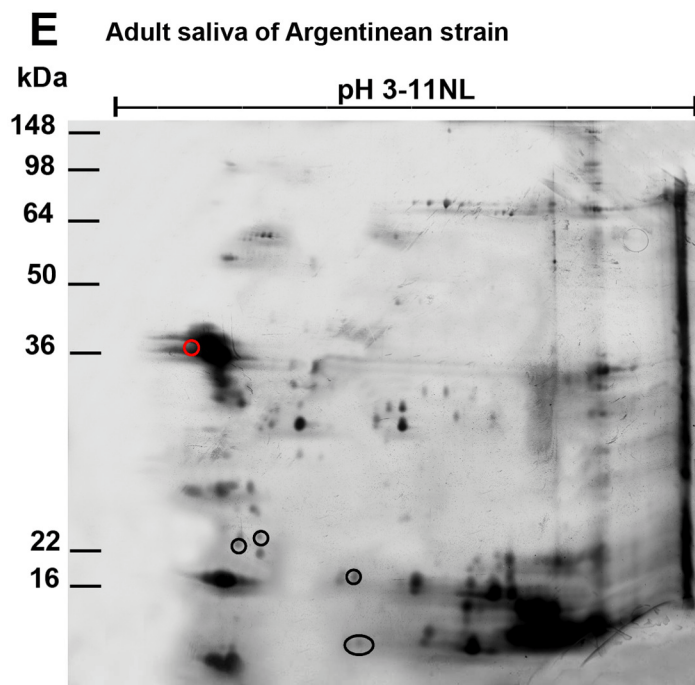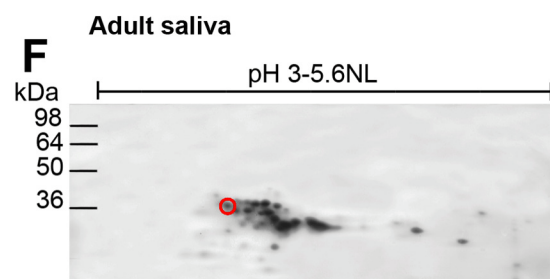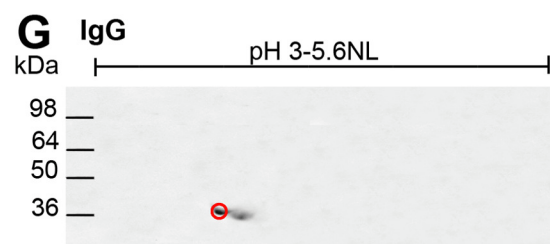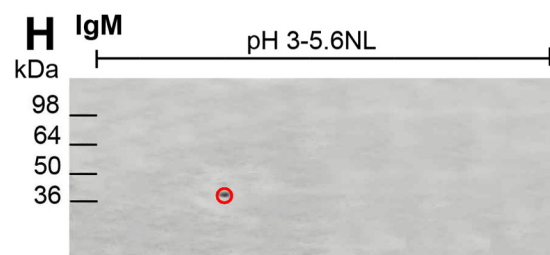

Supplement: Figure S1 — 2D salivary profiles and 2D western blot analyses of nymphal and adult T. infestans from Argentina. Saliva of 5th instar nymphs (A) and adults (females and males, E) of Argentinean T. infestans were isoelectrically focused in the nonlinear pH range of 3–11. Black circles indicate nymphal (A) or adult (E) specific salivary proteins. In order to improve the protein separation, saliva of nymphs (B) and adults (F) were isoelectrically focused in the nonlinear pH range of 3–5.6 and blotted onto nitrocellulose. Comparing IgG (C, G) and IgM antibody reactions (D, H) with salivary proteins of nymphs (C, D) and adults (G, H), the candidate exposure marker antigen of 35 kDa was recognized by IgG and IgM antibodies of guinea pig serum from the 5th week of exposure to the Argentinean T. infestans strain. This protein is marked with a red circle in the different panels. (PDF) [file pntd.0002783.s001.pdf]

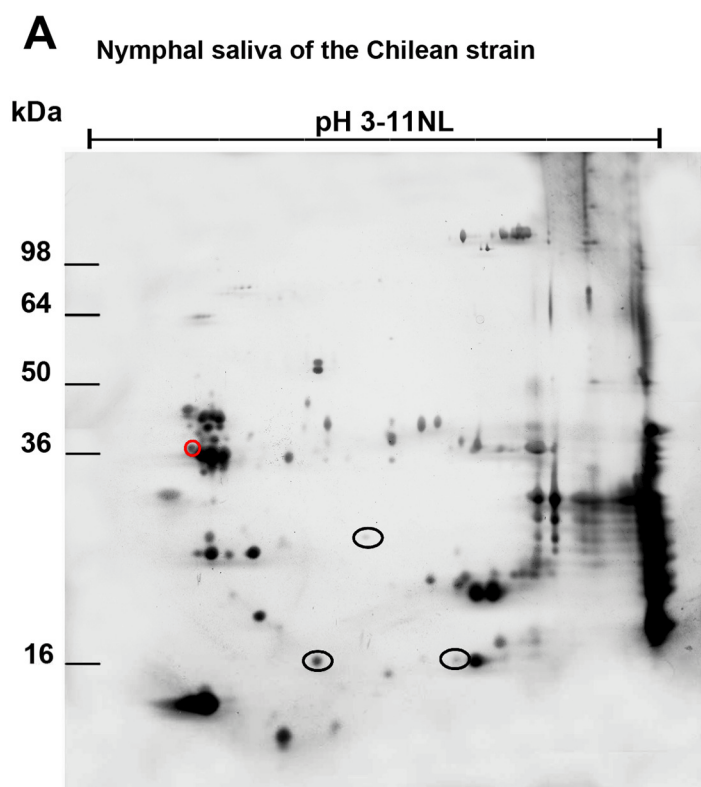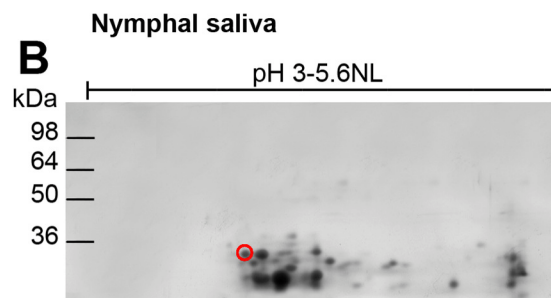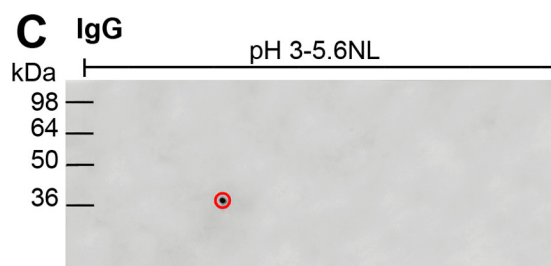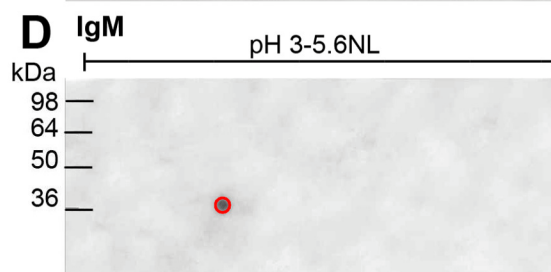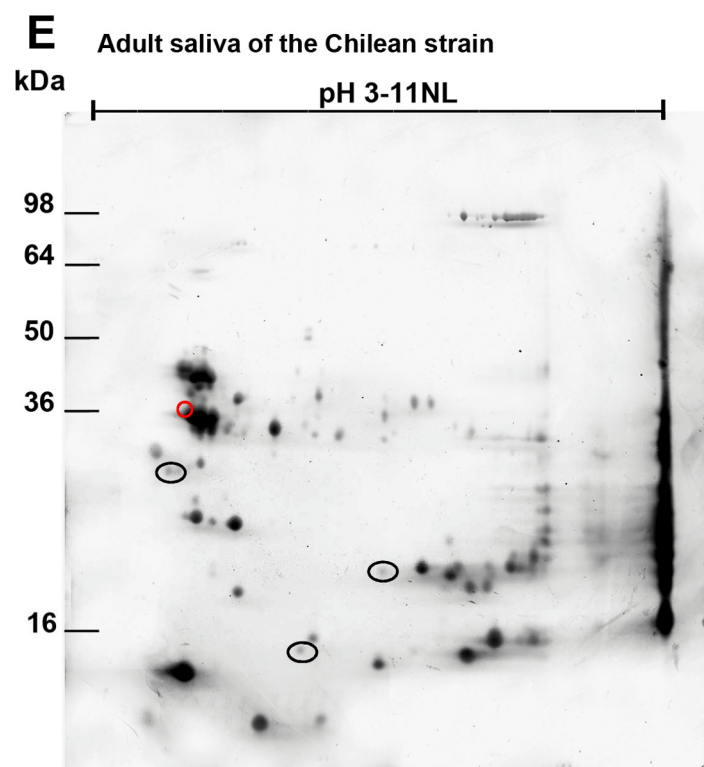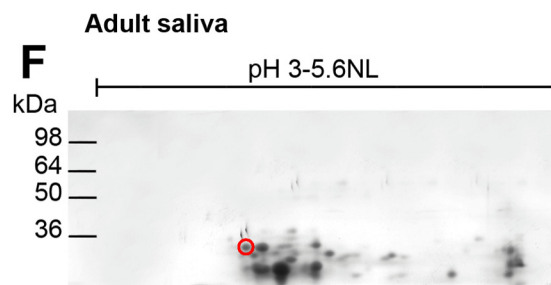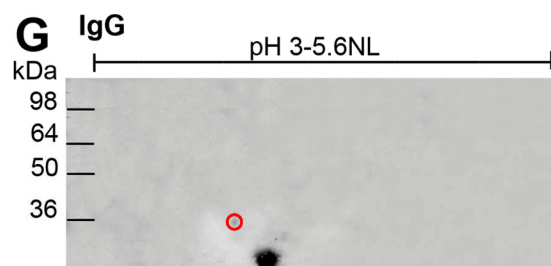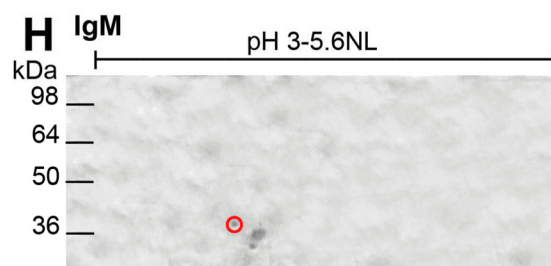

Supplement: Figure S2 — 2D salivary profile and western blot analyses of nymphal and adult T. infestans from Chile. Saliva of 5th instar nymphs (5th instar, A) and adults (females and males, E) of Chilean T. infestans were isoelectrically focused in the nonlinear pH range of 3–11. Black circles indicate nymphal (A) or adult (E) specific salivary proteins. In order to improve the protein separation, saliva of nymphs (B) and adults (F) were isoelectrically focused in the nonlinear pH range of 3–5.6 and blotted onto nitrocellulose. Comparing IgG (C, G) and IgM antibody reactions (D, H) with salivary proteins of nymphs (C, D) and adults (G, H) T. infestans saliva, the candidate exposure marker antigen of 35 kDa was recognized by IgG and IgM antibodies of guinea pig serum from the 5th week of exposure to the Chilean T. infestans strain. This protein is marked with a red circle in the different panels. (PDF) [file pntd.0002783.s002.pdf]
