# Supplementary material for: Characterization of Guinea Pig Antibody Responses to Salivary Proteins of Triatoma infestans for the Development of a Triatomine Exposure Marker
Source: PLoS Negl Trop Dis. 2014 Apr 3;8(4):e2783. doi: 10.1371/journal.pntd.0002783 (PMC3974673; doi:10.1371/journal.pntd.0002783)
Supplement: Table S1 — Origin of different Triatoma infestans strains. (PDF) [file pntd.0002783.s004.pdf]

Table S1. Origin of the *Triatoma infestans* strains.

Origin	Department	Province	Municipality	Community	Habitat	GPS data
Argentina	Libertador General San Martin	Chaco	Pampa del Indio	Pampa del Indio	Peridomestic	26°02'56.47"S, 59°56'32.44"W, 97m
Bolivia	Cochabamba	Capinota	Lipez	Lipez city	Peridomestic	17°34′21.38"S, 66°15′52.93″W, 2525m
Chile	Region of Atacama	Huasco	Vallenar	Cachiyuyo	Domestic	29°02'11.54"S, 70°53'55.08"W, 829 m
Peru	Arequipa	Arequipa	Mariano Melgar	Mariano Melgar	Domestic and peridomestic	16°24' 02.18"S, 71°29'59.13"W, 2464 m
