# Supplementary material for: Characterization of Guinea Pig Antibody Responses to Salivary Proteins of Triatoma infestans for the Development of a Triatomine Exposure Marker
Source: PLoS Negl Trop Dis. 2014 Apr 3;8(4):e2783. doi: 10.1371/journal.pntd.0002783 (PMC3974673; doi:10.1371/journal.pntd.0002783)
Supplement: Table S2 — Immunogenic salivary antigens of four different T. infestans strains. (PDF) [file pntd.0002783.s005.pdf]

Table S2. Immunogenic salivary antigens of four different *T. infestans* strains.

	IgG antigens								IgM antigens							
	Argentina		Bolivia		Chile		Peru		Argentina		Bolivia		Chile		Peru	
**kDa***	**N**	**A**	**N**	**A**	**N**	**A**	**N**	**A**	**N**	**A**	**N**	**A**	**N**	**A**	**N**	**A**
**91**	x						x	x								
**88**		x				x	x	x				x				
**79**	x		x	x	x		x		x	x						
**71**	x	x					x				x	x				
**64**		x				x							x		x	
**60**					x						x	x		x		
**55**		x												x	x	
**51**					x											
**48**						x										
**43**			x	x			x	x			x					
**40**		x				x	x	x							x	x
**36**			x	x		x	x	x				x	x		x	x
**35**	x	x	x	x	x	x	x	x	x	x	x	x	x	x	x	x
**33**	x	x	x	x	x	x	x	x							x	x
**32**				x	x	x	x	x	x					x	x	x
**30**	x		x	x		x	x	x								
**28**	x			x		x									x	
**27**				x												
**25**	x	x	x	x	x	x			x				x	x		
**24**			x	x												
**21**	x			x	x	x		x					x			
**18**					x				x							
**15**			x	x	x	x			x	x				x		
**14**	x		x	x	x											
**13**					x	x										
**12**								x								
**8**						x										

* The presented proteins reacted with IgG or IgM antibodies of at least one guinea pig serum, respectively.
