# Supplementary material for: Characterization of Guinea Pig Antibody Responses to Salivary Proteins of Triatoma infestans for the Development of a Triatomine Exposure Marker
Source: PLoS Negl Trop Dis. 2014 Apr 3;8(4):e2783. doi: 10.1371/journal.pntd.0002783 (PMC3974673; doi:10.1371/journal.pntd.0002783)
Supplement: Table S3 — Correlation between sum of feeding T. infestans and IgG antibody response of guinea pigs. (PDF) [file pntd.0002783.s006.pdf]

Table S3. Correlation between sum of feeding *T. infestans* and IgG antibody response of guinea pigs

**IgG antibody response to saliva of certain developmental stage/strain of *T. infestans***	**Spearman rank correlation test**
Bolivian nymphs	r= 0.988; p<0.0001
Bolivian adults	r= 0.952; p<0.001
Chilean nymphs	r= 0.988; p<0.0001
Chilean adults	r= 0.952; p<0.001
Argentinean nymphs	r= 1.0; p<0.0001
Argentinean adults	r= 0.976; p<0.0001
Peruvian nymphs	r= 0.945; p<0.0001
Peruvian adults	r= 0.951; p< 0.0001
